# Applicability of the Spoken Knowledge in Low Literacy Patients with Diabetes in Brazilian elderly

**DOI:** 10.1590/S1679-45082016AO3747

**Published:** 2016

**Authors:** Jonas Gordilho Souza, Daniel Apolinario, José Marcelo Farfel, Omar Jaluul, Regina Miksian Magaldi, Alexandre Leopold Busse, Flávia Campora, Wilson Jacob-Filho

**Affiliations:** 1 Hospital das Clínicas, Faculdade de Medicina, Universidade de São Paulo, São Paulo, SP, Brazil.; 2 Faculdade de Medicina, Universidade de São Paulo, São Paulo, SP, Brazil.

**Keywords:** Diabetes mellitus, Blood glucose, Survey and questionnaires, Health knowledge, attitudes, practice, Aged

## Abstract

**Objective:**

To translate, adapt and evaluate the properties of a Brazilian Portuguese version of the Spoken Knowledge in Low Literacy Patients with Diabetes, which is a questionnaire that evaluate diabetes knowledge.

**Methods:**

A cross-sectional study with type 2 diabetes patients aged ≥60 years, seen at a public healthcare organization in the city of Sao Paulo (SP). After the development of the Portuguese version, we evaluated the psychometrics properties and the association with sociodemographic and clinical variables. The regression models were adjusted for sociodemographic data, functional health literacy, duration of disease, use of insulin, and glycemic control.

**Results:**

We evaluated 129 type 2 diabetic patients, with mean age of 75.9 (±6.2) years, mean scholling of 5.2 (±4.4) years, mean glycosylated hemoglobin of 7.2% (±1.4), and mean score on Spoken Knowledge in Low Literacy Patients with Diabetes of 42.1% (±25.8). In the regression model, the variables independently associated to Spoken Knowledge in Low Literacy Patients with Diabetes were schooling (B=0.193; p=0.003), use of insulin (B=1.326; p=0.004), duration of diabetes (B=0.053; p=0.022) and health literacy (B=0.108; p=0.021). The determination coefficient was 0.273. The Cronbach a was 0.75, demonstrating appropriate internal consistency.

**Conclusion:**

This translated version of the Spoken Knowledge in Low Literacy Patients with Diabetes showed to be adequate to evaluate diabetes knowledge in elderly patients with low schooling levels. It presented normal distribution, adequate internal consistency, with no ceiling or floor effect. The tool is easy to be used, can be quickly applied and does not depend on reading skills.

## RESUMO

**Objetivo:**

Traduzir, adaptar e avaliar as propriedades de uma versão, em português do Brasil, do *Spoken Knowledge in Low Literacy Patients with Diabetes*, um questionário que avalia conhecimento em diabetes.

**Métodos:**

Estudo transversal, em diabéticos tipo 2, com idade ≥60 anos de uma instituição pública de saúde, em São Paulo (SP). Após o desenvolvimento da versão na língua portuguesa, foram avaliadas suas propriedades psicométricas e associação com variáveis sociodemográficas e clínicas. Os modelos de regressão foram ajustados para dados sociodemográficos, alfabetismo funcional em saúde, tempo de doença, uso de insulina e controle glicêmico.

**Resultados:**

Foram avaliados 129 diabéticos, com média de idade de 75,9 (±6,2) anos, escolaridade média de 5,2 (±4,4) anos, hemoglobina glicada média de 7,2% (±1,4) e valor médio do *Spoken Knowledge in Low Literacy Patients with Diabetes* de 42,1% (±25,8). No modelo de regressão, as variáveis associadas de forma independente ao *Spoken Knowledge in Low Literacy Patients with Diabetes* foram escolaridade (B=0,193; p=0,003), uso de insulina (B=1,326; p=0,004), tempo de doença (B=0,053; p=0,022) e alfabetismo em saúde (B=0,108; p=0,021). O coeficiente de determinação foi de 0,273. O a de Cronbach apresentou valor de 0,75, revelando consistência interna adequada.

**Conclusão:**

Esta versão traduzida do *Spoken Knowledge in Low LiteraFcy Patients with Diabetes* mostrou-se adequada para avaliar conhecimentos em diabetes em idosos de baixa escolaridade, apresentando distribuição normal, consistência interna adequada, sem a presença de efeito teto ou chão. O instrumento teve boa aplicabilidade, já que pôde ser administrado de maneira rápida e não depende da capacidade de leitura.

## INTRODUCTION

Diabetes is a chronic degenerative disease commonly observed in elderly, and its prevalence has increased fastly in recent decades.^(1)^ Strong evidence demonstrated the need of appropriate glycemic control to prevent micro- and macrovascular complications.^(2,3)^ Inadequate glycemic control in adults is due to cognitive impairment, sensory loss, polypharmacy, depression and poor compliance.^(4-6)^

Management of type 2 diabetes also involves learning about the disease and adopting self-care.^(7)^ In this context, the individuals with limited educational background and insufficient health literacy tend to present more difficulties.^(8,9)^

Several tools designed to evaluate diabetes knowledge have been developed in the last decades. Unfortunately most of these involve reading ability and are based on complex scales, with limited clinical applicability, especially for individuals with low schooling level.^(10,11)^

Rothman et al. developed the Spoken Knowledge in Low Literacy Patients with Diabetes (SKILLD),^(12)^ a tool originally designed in English to assess knowledge on this disease. Since SKILLD is applied verbally, it tests parameters that are independent of reading capacity. The questions are written in simple language and the level of difficulty has been adapted for individuals with low schooling level. Furthermore, the open questions allow the individuals to explain their answers in their own words.

Since its publication, SKILLD had neither been adapted to Portuguese language nor used as a questionnaire for elderly people in Brazil, a country where the population aged over 60 years presents low schooling levels.^(13)^

## OBJECTIVE

To translate, adapt and assess the psychometric properties of the Spoken Knowledge in Low Literacy Patients with Diabetes.

## METHODS

### Participants

We recruited individuals treated at the reference outpatient clinic for elderly at the *Hospital das Clínicas da Faculdade de Medicina da Universidade de São Paulo* (FMUSP), between June 2011 and July 2012. We enrolled the individuals who had enough time available to answer the questions. A total of 225 type 2 diabetics aged ≥60 years were assessed. All individuals received free medications from the pharmacy of the organization. None of the participants was participating in diabetes education programs, and all had access to the same healthcare services.

One researcher talked to participants in the waiting room, where they waited for routine medical appointment with geriatricians or residents under training at the outpatient clinic. After accepting the oral invitation to participate in the study, the participants were taken to another room, away from external factors that could impact on their attention and concentration, to answer the questions. All information obtained in the study was classified. The same researcher interviewed all participants.

The inclusion criteria were age ≥60 years, diagnosis of type 2 diabetes under treatment with oral medication or insulin,^(7)^ glycosylated hemoglobin (HbA1c) measured during the last six months, and oral fluency in Portuguese. We excluded individuals with diagnosis of dementia, based on the medical chart report, and those with visual, auditory, motor or language impairment that hindered interaction with the examiner. The situations that could affect accuracy of HbA1C measurement were additional exclusion criteria: hemoglobin <11mg/dL; thyroid stimulating hormone (TSH) <0.1 or >10mU/L. and glomerular filtration rate estimated by Cockcroft-Gault formula <30mL/min/1.73m^2^.^(14-16)^

Furthermore, we also excluded participants who presented frailty syndrome, because less aggressive glycemic control targets have been proposed for this group.^(7,17)^ For this purpose, we used the criteria described in the Study of Osteoporotic Fracture (SOF),^(18)^ which include more than 5% weight loss in the previous year; inability to sit on a chair and stand up five times in a row; reported feeling of lack of energy assessed by question: “Do you feel full of energy?”.

### Spoken Knowledge in Low Literacy Patients with Diabetes

This tool consists of ten questions associated to diabetes knowledge. Only complete answers are accepted and the score varies between zero and 100%. Higher scores are associated with better understanding of the disease. At first, the participant is given 10 to 15 seconds before answering each question. Should the participant fail to answer at this first attempt, the question is reformulated for easier understanding, asked again and further 15 seconds are given for answer. The total time to answer the questionnaire varies from 5 to 10 minutes.^(12)^

The Brazilian version of the SKILLD was independently translated and adapted by two native Brazilian physicians who are fluent in Portuguese and English. The physicians were aware of the aims of the study and had good knowledge of the target population this tool would be applied to. A third physician, who is an experienced member of the group for discrepancy resolution, revised both translations and produced a final version. A pilot study was conducted to test the final version in ten diabetic subjects, who were chosen by convenience to identify possible language and cultural problems. This pilot study yielded satisfactory results revealing that the tool required no changes.

### Sociodemographic and clinical data

The following sociodemographic data were obtained: age, years of schooling, race, marital status (married *versus* non-married), previous occupation (blue collar worker or not). The socioeconomic classes were assessed according to the Criterion for Economic Classification Brazil (CCEB - *Critério de Classificação Econômica Brasil*),^(19)^ which provides a continuous scale that is calculated based on the scores associated with the amount of household items and the schooling level of the person with the highest income in the house. Based on the score, we obtained assessment intervals, which are classified into five subgroups: A (35 to 46), B (23 to 34), C (14 to 22), D (8 to 13) and E (zero to 7).

Participants were also assessed on the duration of diabetes, type of treatment (oral medication or insulin therapy) and assistance with medication (help to obtain, organize, remember when to take medication, and those who were totally dependent).

The most recent HbA1C measurement taken within the last six months was used to assess control of diabetes. Therefore, we used the high performance liquid chromatography (HPLC), and the results were extracted from the patient’s electronic file available at the service. For the purpose of this study, glycemic control was considered inadequate when HbA1c ≥7%.

Previous studies demonstrated that depressive symptoms may influence glycemic control.^(5)^ To assess depressive symptoms we used the Geriatric Depression Scale with 15 items (GDS-15). The GDS-15 is a questionnaire with dichotomous answers (yes or no) and provides a continuous measure of severity. We used the version that had been validated for Brazil, comprising 15 items. A score ≥5 indicates depression.^(20)^

For the assessment of functional health literacy we used the Short Assessment of Health Literacy for Portuguese-speaking Adults (SAHLPA-18),^(21)^ which is a tool validated for Portuguese that measures understanding and pronunciation of relatively common medical words.

During the literacy assessment cards are used. On the top of each card, a medical term is written in bold letters. At the bottom of the card, there are two additional words, but only one is associated with the word on the top of the card. At first, the interviewee is asked to read out loud the word on the top of the card. Then the interviewer reads out aloud the words on the bottom of the card, and asks which is associated with the medical term. Each individual is given a set of 18 cards, and a score is attributed to each item considering correct association and pronunciation. The final score varies from zero to 18. Individuals who score ≤14 are classified as people with insufficient health literacy.

### Statistical analysis

The description of interval variables included mean and standard deviation. After normality assessment test, based on histogram graphs, we carried out a bivariate analysis with parametric statistics.

In order to evaluate the association between the level of knowledge about diabetes with sociodemographic and clinical characteristics, the participants were divided into two groups: adequate knowledge (SKILLD >50%) and inadequate (SKILLD ≤50%).^(20)^ To compare category variables between these two groups we used the χ^2^ test. For comparison of interval variables, the Student´s *t* test for independent samples was employed.

The SKILLD score was also evaluated according to intervals. Univariate and multivariate regression models were created to investigate the associated factors regardless of the level of knowledge on diabetes. According to this model, the SKILLD score was defined as a dependent variable. The explanatory variables considered for insertion in this model were age, sex, schooling, insufficient functional literacy, marital status, economic level, duration of disease, use of insulin, and glycemic control. We used the backward strategy to insert variables that had a significance level <0.1 in the bivariate analysis and those with great clinical relevance in relation to SKILLD.

In order to evaluate the internal consistency of the tool we used Cronbach’s α*.* To determine the properties of each item we calculated adjusted item-total correlation, changes in Cronbach after removal of each item, point biserial correlation coefficient associated with schooling and SAHLPA-18. To investigate the factorial structure of the instrument, we carried out an analysis of the main components in a tetrachoric correlation matrix. The sedimentation graph is derived from the self-scores attributed to visual inspection, and Horn’s parallel analysis was conducted to compare the dimension of self-scores obtained by the analysis of main components with those obtained by 100 random samples.

Two-tailed test were used to assess data. A p value of 0.05 was considered statistically significant. The analysis were carried out using Statistical Package Social Sciences (SPSS) version 20.0 and Stata version 13.0

This protocol was approved by the Research Ethics Committee of the organization and is registered under 0534/11. All participants signed an Informed Consent Form. In the case of illiterate participants, the form was read out and explained aloud to them, in the presence of an impartial witness. The form was signed by the participant, or by the legal representative.

## RESULTS

We invited 225 type 2 diabetic individuals to participate in the study. A total of 90 were excluded for presenting one of the following characteristics: dementia (51), frailty syndrome (13), severe visual impairment (10), severe hearing impairment (1), renal failure (9), lack of fluency in Portuguese (3), anemia (2), aphasia (1). Of the remaining individuals, six refused to participate in the study. Thus, a total of 129 participants were included in the analysis.

Table 1 displays the sociodemographic and clinical characteristics according to level of knowledge about diabetes. The total sample yielded a mean age of 75.9 (±6.2) years, and 69.8% were female. Average schooling was 5.2 (±6.2) years, and 82.9% had not completed junior school. According to the SAHLPA-18 score, 56.6% of participants were classified as inadequate health literate, with a mean score of 12.1 (±5.3). Average duration of diabetes was 12.8 years (±9.1), and 31.8% of participants were on insulin therapy. The mean glycosylated hemoglobin value was 7.2% (±1.4).

**Table 1:** Sociodemographic and clinical characteristics: comparison between groups with sufficient and insufficient knowledge

Characteristics	Total sample	Sufficient knowledge	Insufficient knowledge	p value
	(n=129)	(n=42)	(n=87)	
Age, years	75.9 (6.2)	75 (6.9)	76.4 (5.8)	0.230*
Sex, female	90 (69.8)	26 (61.9)	64 (73.6)	0.177^†^
Race, White	61 (47.3)	18 (42.9)	43 (49.4)	0.484^†^
Schooling, years	5.2 (4.4)	6.6 (5.1)	4.5 (3.8)	0.011*
Socioeconomic class (CCEB)	20 (6.2)	20.4 (5.8)	19.7 (6.4)	0.539*
Blue-collar worker	62 (48.1)	18 (42.8)	44 (50.6)	0.411^†^
Marital status, married	42 (32.6)	16 (38.1)	26 (29.9)	0.351^†^
Help with medication	22 (17.1)	8 (19.1)	14 (16.1)	0.676^†^
Symptoms of depression (GDS-15)	3.4 (2.7)	2.8 (2.4)	3.6 (2.8)	0.113*
Use of Insulin	41 (31.8)	20 (47.6)	21 (24.1)	0.007^†^
Duration of diabetes. years	12.8 (9.1)	16.5 (8.8)	11.1 (8.7)	0.001*
Health literacy (SAHLPA-18)	12.1 (5.3)	13.7 (4.8)	11.34 (5.4)	0.01*
HbA1c	7.2 (1.4)	7.5 (1.4)	7.08 (1.4)	0.073*

*Student´s *t* test for independent samples comparing sufficient and insufficient knowledge; ^†^ χ^2^ test comparing adequate and inadequate knowledge. Adequate knowledge corresponded to Spoken Knowledge in Low Literacy Patients with Diabetes >50%; inadequate knowledge to Spoken Knowledge in Low Literacy Patients with Diabetes ≤50%. Results expressed in n (%) or mean (± standard deviation). CCEB: *Critério de Classificação Econômica Brasil*; SAHLPA-18: *Short Assessment of Health Literacy for Portuguese-speaking Adults-*18; GDS-15: *Geriatric Depression Scale* with 15 questions; HbA1c: glycosylated hemoglobin.

### Variables associated with Spoken Knowledge in Low Literacy Patients with Diabetes

In assessing the differences between both groups classified according to SKILLD regarding the level of knowledge about diabetes (sufficient *versus* insufficient), we observed a statistically significant difference in schooling level (6.6 (±5.1) *versus* 4.5 (±3.8); p=0.011); mean SAHLPA-18 (13.7 (±4.8) *versus* 11.3 (±5.4); p=0.016); use of insulin (47.6% *versus* 24.1%; p=0.007); and duration of diabetes (16.5 years (±8.8) *versus* 11.1 (±8.7); p = 0.001) (Table 1). The univariate regression analysis demonstrated the following variables were associated with SKILLD: schooling level (B=0.232; p<0.001); marital status - married (B=0.483; p=0.031); use of insulin (B=1.432; p=0.003); duration of diabetes (B=0.071; p=0.005) and SAHLPA-18 (B=0.182; p<0.001) (Table 2).

**Table 2:** Association of sociodemographic and clinical characteristics in a univariate and multivariate linear model to predict the score of the Spoken Knowledge in Low Literacy Patients with Diabetes

Characteristics	Without adjustment		With adjustment*	
	
	Coefficient	p value	Coefficient	p value
Age, years	-0.070	<0.063		
Sex, female	-0.825	<0.099		
Race, White	-0.305	<0.509		
Schooling, years	0.232	<0.001	0.193	0.003
Socioeconomic class (CCEB)	0.070	<0.063		
Blue-Collar Worker	-0.870	<0.058	0.430	0.350
Marital status, married	0.483	<0.031		
Help with medication	-0.538	<0.469		
Symptoms of depression (GDS-15)	-0.095	<0.265		
Use of insulin	1.432	<0.003	1.326	0.004
Duration of diabetes, years	0.071	<0.005	0.053	0.022
Health literacy (SAHLPA-18)	0.182	<0.001	0.108	0.021
HbA1c	0.248	<0.138		

R^2^=0.273; p<0.001. adjusted for: scholling, blue collar worker, use of insulin, duration of diabetes, SAHLPA-18 score; Variance analysis test (ANOVA) to compare statistical significance of the models. CCEB: *Critério de Classificação Econômica Brasil*; GDS-15: Geriatric Depression Scale 15 questions; SAHLPA-18: Short Assessment of Health Literacy for Portuguese-speaking Adults-18; HbA1c: glycosylated hemoglobin.

In the multivariate linear regression model corrected for blue-collar work, the following variables were independently associated with SKILLD: schooling (B=0.193; p=0.003), use of insulin (B=1.326; p=0.004), duration of diabetes (B=0.053; p=0.022), and SAHLPA-18 score (B=0.108; p=0.021). The determination coefficient was 0.273, and p<0.001 (Table 2).

### Psychometric properties of Spoken Knowledge in Low Literacy Patients with Diabetes

Spoken Knowledge in Low Literacy Patients with Diabetes presented normal distribution and mean value of 42.1% (±25.8%). The final translated model including ten questions and answers is described in chart 1. The questions with the highest percentage of right answers were: “what is the treatment for low glycemia?”, 65.1%; and “how often should a diabetic individual see an eye doctor and why is this important?”, 66.7%. The questions with the lowest percentage of right answers were: “what are the signs and symptoms of high glycemia?”, 23.3%; and “which is the normal value of glycosylated hemoglobin?”, 10.9%.

Cronbach’s α coefficient was 0.75, revealing adequate internal consistency. As shown on table 3, nine out of ten questions presented question-total correlation ≥0.4, which indicates good discrimination power. The question associated with correct frequency of physical exercise was the only one that presented a lower question-total correlation (0.20), and the only one that resulted in an increase in Cronbach’s coefficient when removed (0.76).

**Table 3:** Evaluation of each question of the Spoken Knowledge in Low Literacy Patients with Diabetes and its correlation with schooling and health literacy

SKILLD answer	Proportion of right answers (%)	Adjusted item-total correlation	Change in Cronbach	Correlation of biserial point and schooling	Correlation of biserial point and SAHLPA-18
Hyperglycemia symptoms	0.23	0.40	0.74	0.14*	0.08
Hypoglycemia symptoms	0.30	0.45	0.73	0.06*	0.10
Hypoglycemia treatment	0.65	0.50	0.72	0.22*	0.26^†^
Frequency of feet examination	0.35	0.44	0.73	0.28^†^	0.23^†^
Reason for feet examination	0.49	0.53	0.72	0.30^†^	0.33^‡^
Frequency of eye examination	0.67	0.46	0.73	0.28^†^	0.30^‡^
Normal values of fasting glycemia	0.55	0.43	0.73	0.28^†^	0.35^‡^
Normal values of glycosylated hemoglobin	0.11	0.40	0.74	0.47^‡^	0.22*
Exercise frequency	0.31	0.20	0.76	0.07*	0.04
Long-term complications	0.54	0.40	0.74	0.19*	0.17

* p value <0.05; ^†^ p value <0.01; ^‡^ p value <0.001. SKILLD: Spoken Knowledge in Low Literacy Patients with Diabetes; SAHLPA-18: Short Assessment of Health Literacy for Portuguese-speaking Adults-18.

**Figure 1:**
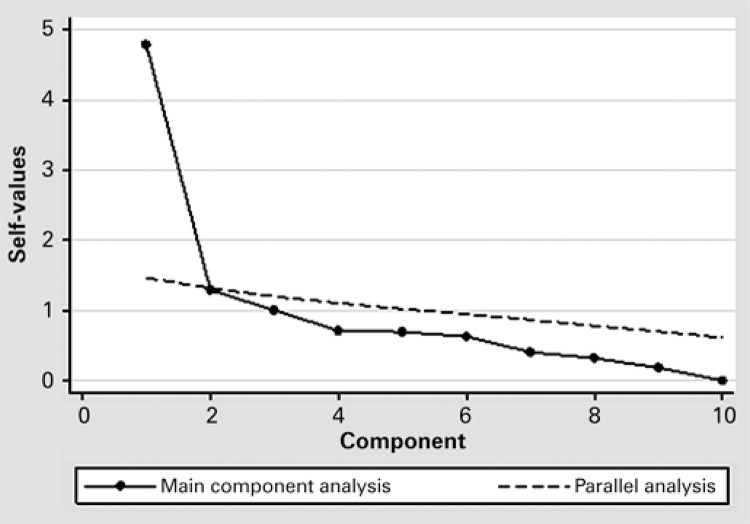
Horn’s parallel analysis applicability of the Spoken Knowledge in Low Literacy Patients with Diabetes in Brazilian elderly

The main component analysis revealed two factors with self-value above 1, the first of 4.79, explaining 47.9% of variance, and the second of 1.29, explaining 13% of additional variance. Visual inspection suggested a one-dimensional structure. Horn’s parallel analysis revealed only one factor with a self-value greater than the mean corresponding to random samples (4.79 *versus* 1.46), confirming the one-dimensional nature of the tool (Figure 1).

## DISCUSSION

The SKILLD score proved to be appropriate for this sample of elderly people. It presented normal distribution, adequate internal consistency and with no ceiling and floor effect. The SKILLD has some advantages when compared to other instruments used to evaluate diabetes knowledge. Since it is a simple oral test, it is easy to use in individuals with low schooling level. Moreover, other tools include questions associated with the pathophysiology of the disease, which do not seem to be essential for adequate self-care.^(10,11,22)^

The frequency of errors observed in the SKILLD reflects the low schooling of the sample population. Other authors who evaluated individuals with low schooling observed a similar proportion of right and wrong answers related to the topics of the questionnaire.^(12,23)^ In our study, 82.9% of participants had incomplete high school. This percentage is even higher than that observed in the SKILLD validation study, where 40% of participants had incomplete high school.^(12)^

Only 10.9% of individuals answered correctly the question about the ideal value of glycosylated hemoglobin. The correct answer to this question was glycosylated hemoglobin should be below 7.0%. Nevertheless, it is important to point out that current guidelines suggest that this cutoff point is not appropriate for all elderly individuals, because there are risks associated with intense glycemic control, especially in more fragile individuals, who present loss of functional capacity.^(7,17,24)^

We also observed that some participants had trouble answering the question about exercise, because according to them exercise should be more often and more intense than indicated. The analysis of the psychometric properties of the score revealed that this question presents low item-total correlation and that withdrawing it would increase internal consistency, suggesting that this question is of limited use and could possibly be excluded.

Regression model analysis showed an association between SKILLD and schooling, use of insulin, duration of disease and functional health literacy. The determination coefficient of the final model was 0.273, which indicates that the variation in SKILLD is for the most part explained by factors that were not represented in this model. Despite the fact that SKILLD is an oral test, schooling and literacy influenced the results, because these individuals probably have less access to information.^(25)^ Rothman et al., reported similar results, and observed a correlation between the SKILLD and the variables schooling level, health literacy, duration of disease, and use of insulin. Contrary to our results, these authors also observed a negative correlation between HbA1c levels and SKILLD score. It is important to point out that the validation study did not test the association of each variable and the results in an independent manner.^(12)^

We must also mention some limitations of our study. It was not possible to establish causality in the relations between SKILLD and other variables, for being a cross sectional study. We underline the absence of criterion validation, since the translated SKILDD version has not been compared with another questionnaire about diabetes knowledge. This study did not allow evaluation of this criterion, because most tools that measure diabetes knowledge were originally described in English, and the instruments validated in Brazil have not been adjusted for low-schooling populations, for involving reading ability and complex questions on pathophysiology of disease.^(25)^ Neither did we evaluate reliability parameters, such as inter-examiner agreement and test-retest stability. Another limitation of the study was the subjective influence of the professional when scoring the answer of participants. In order to avoid measurement biases, the same researcher carried out all SKILLD evaluations.

The participants of our study were recruited by convenience, in a single tertiary care hospital, which limits generalization of results. Some factors that might be associated with knowledge on diabetes were not included in this study, and must be taken into account in future studies. Among these factors, we include cognitive performance, complexity of therapeutic regimen, diabetes education, motivation and attitude regarding treatment.^(4,7,17)^

Further studies must be carried out to compare SKILLD with other questionnaires on diabetes knowledge, and to assess the inter-examiner and test-retest reliability.

## CONCLUSION

The Portuguese version of Spoken Knowledge in Low Literacy Patients with Diabetes is adequate to evaluate diabetes knowledge in elderly patients with low schooling. This tool is easy to use, can be quickly applied and does not depend on reading ability of patients. In our sample, Spoken Knowledge in Low Literacy Patients with Diabetes was independently associated with the following variables: schooling, use of insulin, duration of disease and functional health literacy.

**Chart 1:** Spoken Knowledge in Low Literacy Patients with Diabetes, Portuguese translation

Question	Answer
Q1. Which are the signs and symptoms of high glycemia? What does a diabetic person feel when the level of sugar in the blood is very high?	At least two: excessive thirst, frequent urination, drink a lot of liquids, eat too much, blurred vision, dizziness/weakness
Q2. Which are the signs and symptoms of low glycemia? What does a diabetic person feel when the level of sugar in the blood is very low?	At least two: hunger, nervous/agitated, mood swings/irritable, confused, excessive sweating, accelerated heart beat
Q3. What treatment should be given in cases of low glucose levels? What should you do when the level of sugar in the blood drop a lot? What should you do to increase the amount of sugar in the blood when it is very low?	Accept general answers: drink juice/milk eat sweets/15 g of carbohydrates
Q4. How often should a diabetic individual examine their feet? Once a day? Once a week? Once a month?	Accept only: daily
Q5. Why is it important that diabetic individuals examine their feet? Why should you examine your feet? What should you look for?	Accept general answers: prevention or detection of problems caused by complications of diabetes.
Q6. How often should a diabetic individual see an eye doctor, and why is this important? How often?	Accept at least once a year AND to diagnose/treat problems of the retina, glaucoma, blindness.
Q7. Which is the normal fasting glycaemia? When the person wakes up and checks the level of sugar in the blood, before eating or taking medication, it should be between which values? Which is the normal range for fasting glycaemia?	Accept variation between 70-80 to 100-120
Q8. Which is the normal value for glycosylated hemoglobin? When a person collects blood to determine the average level of sugar, up to what value is it considered normal?	Accept normal ≤6% or target ≤7%
Q9. How often should a diabetic individual exercise and for how long? How many times a week? How long every day?	Accept 3 to 5 times a week AND 30-45 minutes each time
Q10. Which are the long-term consequences of uncontrolled diabetes? What problem can a diabetic have after some years?	At least two of the following: sight problems, renal problems/dialysis, amputation, neuropathy/impotence/gastroparesis, cardiovascular diseases

## References

[1] Wild S, Roglic G, Green A, Sicree R, King H (2004). Global prevalence of diabetes. estimates for the year 2000 and projections for 2030. Diabetes Care.

[2] UK Prospective Diabetes Study (UKPDS) Group (1998). Intensive blood-glucose control with sulphonylureas or insulin compared with conventional treatment and risk of complications in patients with type 2 diabetes (UKPDS 33). Lancet.

[3] Holman RR, Paul SK, Bethel MA, Matthews DR, Neil HA (2008). 10-year follow-up of intensive glucose control in type 2 diabetes. N Engl J Med.

[4] Kirkman MS, Briscoe VJ, Clark N, Florez H, Haas LB, Halter JB, Huang ES, Korytkowski MT, Munshi MN, Odegard PS, Pratley RE, Swift CS, Consensus Development Conference on Diabetes and Older Adults (2012). Diabetes in older adults: a consensus report. J AM Geriatr Soc.

[5] Papelbaum M, Moreira RO, Coutinho W, Kupfer R, Zagury L, Freitas S (2011). Depression, glycemiccontrol and type 2 diabetes. Diabetol Metab Syndr.

[6] Guillausseau PJ (2005). Impact of compliance with oral antihyperglycemic agentes on health outcomes in type 2 diabetes mellitus: a focus on frequency of administration. Treat Endocrinol.

[7] American Diabetes Association (2016). 7. Approaches to Glycemic Treatment. Diabetes Care.

[8] Souza JG, Apolinario D, Magaldi RM, Busse AL, Campora F, Jacob-Filho W (2014). Functional health literacy and glycaemic control in older adults with type 2 diabetes: a cross-sectional study. BMJ Open.

[9] Schillinger D, Grumbach K, Piette J, Wang F, Osmond D, Daher C (2002). Association of health literacy with diabetes outcomes. JAMA.

[10] Dunn SM, Bryson JM, Hoskins PL, Alford JB, Handelsman DJ, Turtle JR (1984). Development of the diabetes knowledge (DKN) scales: forms DKNA, DKNB, and DKNC. Diabetes Care.

[11] Speight J, Bradley C (2001). The ADKnowl: identifying knowledge deficits in diabetes care. Diabet Med.

[12] Rothman RL, Malone R, Bryant B, Wolfe C, Padgett P, DeWalt DA (2005). The Spoken Knowledge in Low Literacy in Diabetes Scale: a diabetes knowledge scale for vulnerable patients. Diabetes Educ.

[13] Instituto Brasileiro de Geografia e estatística (IBGE) (2013). Perfil dos idosos responsáveis pelos domicílios.

[14] Ford ES, Cowie CC, Li C, Handelsman Y, Bloomgarden ZT (2011). Iron-deficiency anemia, non-iron-deficiency anemia and HbA1c among adults in the US. J Diabetes.

[15] Kim MK, Kwon HS, Baek KH, Lee JH, Park WC, Sohn HS (2010). Effects of thyroid hormone on A1C and glycated albumin levels in nondiabetic subjects with overt hypothyroidism. Diabetes Care.

[16] Sharif A, Baboolal K (2010). Diagnostic application of the A(1c) assay in renal disease. J Am Soc Nephrol.

[17] Moreno G, Mangione CM, Kimbro L, Vaisberg E, American Geriatrics Society Expert Panel on Care of Older Adults with Diabetes Mellitus (2013). Guidelines abstracted from the American Geriatrics Society Guidelines for Improving the Care of Older Adults with Diabetes Mellitus: 2013 update. J Am Geriatr Soc.

[18] Ensrud KE, Ewing SK, Taylor BC, Fink HA, Cawthon PM, Stone KL (2008). Comparison of 2 frailty indexes for prediction of falls, disability, fractures, and death in older women. Arch Intern Med.

[19] Associação Brasileira de Empresas de Pesquisa (ABEP) (2008). Critério de Classificação Econômica Brasil.

[20] Paradela EM, Lourenço RA, Veras RP (2005). Validation of geriatric depression scale in a general outpatient clinic. Rev Saude Publica.

[21] Apolinario D, Braga Rde C, Magaldi RM, Busse AL, Campora F, Brucki S (2012). Short Assessment of Health Literacy for Portuguese-speaking Adults. Rev Saude Publica.

[22] Torres HC, Virginia AH, Schallc VT (2005). Validation of Diabetes Mellitus Knowledge (DKN-A) and Attitude (ATT-19) Questionnaires. Rev Saude Publica.

[23] Guler N, Oguz S (2011). The spoken knowledge of low literacy in patients with diabetes. Diabetes Res Clin Pract.

[24] Gerstein HC, Miller ME, Byington RP, Goff DC, Bigger JT, Buse JB, Cushman WC, Genuth S, Ismail-Beigi F, Grimm RH, Probstfield JL, Simons-Morton DG, Friedewald WT, Action to Control Cardiovascular Risk in Diabetes Study Group (2008). Effects of intensive glucose lowering in type 2 diabetes. N Engl J Med.

[25] Carthery-Goulart MT, Anghinah R, Areza-Fegyveres R, Bahia VS, Brucki SM, Damin A (2009). Performance of a Brazilian population on the test of functional health literacy in adults. Rev Saude Publica.

